# Effects of Maxillary Sinus Augmentation on Endosseous Dental Implant Survival: A Systematic Review and Meta-Analysis

**DOI:** 10.7759/cureus.103201

**Published:** 2026-02-08

**Authors:** Ali A Derbishi, Ola Mubarki, Razan M Hakami, Balqees A Alhazmi, Nadia A Swaid, Alanoud M Arim, Ghadeer G Alsulamiy, Shahad H Aldarbi, Bashayr S Alnawmasi, Omar S Khayat, Najoud S Alhawiti, Noura F Alshahrani, Abdulaziz O Hussain, Aban I Younis, Abdullah M Almalki

**Affiliations:** 1 Oral and Maxillofacial Surgery, Sabya General Hospital, Jazan, SAU; 2 College of Dentistry, Jazan University, Jazan, SAU; 3 College of Dentistry, King Khalid University, Abha, SAU; 4 College of Dentistry, Umm Al-Qura University, Makkah, SAU; 5 College of Dentistry, Najran University, Najran, SAU; 6 Dentistry, Private Practice, Hail, SAU; 7 Collage of Dentistry, Taif University, Taif, SAU; 8 General Dentistry, Saudi Arabia Ministry of Health, Tabuk, SAU; 9 Dental Surgery, College of Dentistry, King Faisal University, Al-Ahsa, SAU; 10 Restorative Dentistry, College of Dentistry, Taif University, Taif, SAU; 11 College of Dentistry, Taif University, Taif, SAU

**Keywords:** dental implants, endosseous, implant survival, maxillary sinus augmentation, meta-analysis, sinus lift, systematic review

## Abstract

Maxillary sinus floor augmentation (MSFA) is a procedure for rehabilitating the atrophic posterior maxilla, but the optimal surgical technique and its long-term impact on implant survival remain debated. This systematic review and meta-analysis were conducted to synthesize the available evidence on the long-term survival of endosseous dental implants placed in augmented maxillary sinuses. Following Preferred Reporting Items for Systematic Reviews and Meta-Analyses (PRISMA) guidelines, a search of major electronic databases was performed to identify randomized controlled trials (RCTs) and cohort studies reporting implant survival rates after MSFA with at least one year of follow-up. The primary outcome was implant failure rate. The secondary outcome was marginal bone loss (MBL). Two reviewers independently performed study selection, data extraction, and risk of bias assessment using the Cochrane Risk of Bias 2 (RoB 2) and Risk Of Bias In Non-randomized Studies of Interventions (ROBINS-I) tools. A random-effects model with Hartung-Knapp adjustment was used for meta-analysis, employing the Freeman-Tukey double arcsine transformation for proportions. Subgroup analysis and meta-regression were performed to explore heterogeneity. The certainty of the evidence was assessed using the Grading of Recommendations, Assessment, Development, and Evaluations (GRADE) approach. Twenty-two studies, comprising 6,860 implants, were included. The pooled implant failure rate was 3% (95% confidence interval {CI}: 2-5%), corresponding to a cumulative survival rate of 97%. Significant heterogeneity was observed (I^2^=86.3%). Subgroup analysis revealed a significantly lower failure rate for implants placed via a crestal approach compared to a lateral window approach (p<0.001). The pooled mean MBL was 1.17 mm (95% CI: 0.59-1.75 mm), with very high heterogeneity (I^2^=99.6%). Formal tests did not indicate significant publication bias. The certainty of the evidence for both implant failure and MBL was graded as low. Endosseous dental implants placed in augmented maxillary sinuses demonstrate a high long-term survival rate, confirming the predictability of MSFA. The crestal approach is associated with fewer implant failures, although this association may be influenced by case selection bias. The overall low certainty of the evidence, driven by methodological heterogeneity and the predominance of observational studies, highlights the need for standardized, long-term RCTs to refine clinical protocols and strengthen treatment guidelines.

## Introduction and background

Rehabilitation of the edentulous posterior maxilla is a significant challenge in implant dentistry due to the physiological process of centripetal resorption of the alveolar crest and pneumatization of the maxillary sinus following tooth loss [1,2]. To overcome insufficient vertical bone height (RBH), maxillary sinus floor augmentation (MSFA) has been established as the primary intervention. MSFA protocols are categorized into the following two distinct surgical approaches: the lateral window technique (antrostomy), traditionally reserved for severe atrophy, in which access is gained through the buccal wall; and the crestal (transalveolar) approach, a less invasive osteotome-mediated technique generally applied when greater residual bone exists [3,4]. While advancements in surgical technology, such as piezoelectric bony window osteotomy, have minimized intraoperative complications, such as membrane perforation, the variability in treatment protocols necessitates a rigorous evaluation of their long-term efficacy [5].

The success of MSFA and subsequent implant survival are influenced by the interplay of surgical techniques, grafting materials, and implant characteristics. Autogenous bone is considered the preferred grafting material; however, recent evidence suggests that valid alternatives, such as xenografts, alloplasts, or the use of biologics, such as platelet-rich fibrin (PRF), may offer comparable outcomes with reduced morbidity [6,7]. Furthermore, the concept of graftless sinus elevation, which depends on the space-making effect of the implant and the osteogenic potential of the Schneiderian membrane, has emerged as a viable and less invasive alternative [8]. Systematic reviews have indicated that simultaneous implant placement in non-grafted sinuses can yield high survival rates, challenging the necessity of exogenous graft materials in specific clinical scenarios [8].

The maintenance of marginal bone levels (MBL) is critical for the long-term survival of implants. Factors such as implant design, specifically the distinction between bone- and tissue-level implants, play a pivotal role in preserving crestal bone architecture [9]. Additionally, the preoperative condition of the maxillary sinus and the development of postoperative pathologies are critical determinants. Recent research has highlighted that specific CBCT findings and mucosal thickening must be evaluated to prevent complications that could jeopardize the implant [10].

Therefore, the objective of this systematic review was to determine the long-term survival rates of endosseous dental implants following MSFA. Specifically, this review aimed to compare implant failure rates between lateral window and crestal approaches; quantify MBL as a measure of stability; and assess the impact of heterogeneity regarding graft materials and study design on these outcomes, incorporating data published up to 2025.

## Review

Methods

Protocol and Registration

This systematic review and meta-analysis were conducted in accordance with the Preferred Reporting Items for Systematic Reviews and Meta-Analyses (PRISMA) guidelines [11]. The protocol was prospectively registered in the International Prospective Register of Systematic Reviews (PROSPERO) (#CRD420261277012).

Search Strategy and Data Sources

A search strategy was implemented across major electronic databases (PubMed/MEDLINE, Scopus, Web of Science, and Cochrane Central Register of Controlled Trials {CENTRAL}) to identify studies published from inception to December 2025. The search strategy utilized a combination of controlled vocabulary (MeSH terms) and free-text keywords focused on the concepts of "maxillary sinus floor augmentation," "sinus lift," "dental implants," and "survival rate." To ensure saturation of the literature, the electronic search was supplemented by manual screening of the reference lists of included articles and previously published systematic reviews. The inclusion criteria comprised randomized controlled trials (RCTs) and prospective or retrospective cohort studies with a minimum follow-up of one year post-loading. Studies were excluded if they lacked primary data on implant survival or focused solely on zygomatic or pterygoid implants. Two independent reviewers screened the titles, abstracts, and full texts. Inter-rater reliability during the selection and data extraction phases was quantified using kappa statistics to ensure consistency and minimize selection bias [12].

Quality and Risk of Bias Assessment

The methodological quality of the included studies was rigorously appraised using design-specific tools. The risk of bias for RCTs was evaluated using the Cochrane Risk of Bias 2 (RoB 2) tool [13], while non-randomized studies were assessed using the Risk Of Bias In Non-randomized Studies of Interventions (ROBINS-I) tool [14]. Reporting and dissemination biases were evaluated to identify selective outcome reporting. The assessment of bias related to small-study effects was performed via visual assessment of funnel plots. The asymmetry of these plots was further quantified using Egger’s regression and Begg’s rank correlation tests to detect potential publication bias [15,16].

Data Synthesis and Statistical Analysis

All statistical analyses were performed using R software version 4.5.1 (Vienna, Austria: R Foundation for Statistical Computing) [17]. Due to the anticipated clinical and methodological diversity among studies (e.g., varying graft materials and surgical techniques), a random-effects model was employed to estimate the pooled effects [18].

Effect Measures and Statistical Models

Effect measures were selected based on the outcome type. For dichotomous outcomes (implant survival/failure), risk ratios (RR) or odds ratios (OR) were calculated. For continuous outcomes (marginal bone loss), mean differences (MD) or standardized mean differences (SMD) were utilized. To account for variations in follow-up duration across longitudinal studies, survival analysis specifics included the calculation of incidence rate ratios (IRR) [19].

To address the issue of single-arm survival rates approaching 100%, data transformation using the Freeman-Tukey double arcsine method was applied to stabilize variances and ensure normal distribution prior to pooling [20]. Precision was expressed using 95% confidence intervals (CI). Furthermore, the Hartung-Knapp-Sidik-Jonkman method was applied to adjust the variance estimates and provide more robust confidence intervals, particularly given the potential for heterogeneity [21]. Prediction intervals were calculated to estimate the range in which the effect of a future study would likely fall [22].

Assessment of Heterogeneity and Sensitivity Analysis

Heterogeneity (dispersion) was quantified using the I^2^ statistic and the χ^2^ test for statistical heterogeneity (inconsistency) [23]. To explore the sources of heterogeneity, moderators were analyzed using subgroup analysis (e.g., graft type, surgical approach) and meta-regression for continuous covariates (e.g., residual bone height) [24]. The relationship strength between follow-up time and bone loss was assessed using a correlational analysis. The robustness of the findings was verified through sensitivity analyses (e.g., excluding high-bias studies) and adjustment for confounding variables.

Advanced Assessments and Strength of Evidence

Statistical power (post-hoc analysis) was conducted to determine whether the included studies provided sufficient power to detect significant differences [25]. The temporal evolution of survival rates was examined using a cumulative meta-analysis to observe trends over time [26]. The certainty/strength of evidence for the overall body of literature was graded using the Grading of Recommendations, Assessment, Development, and Evaluations (GRADE) approach, categorizing evidence from high to very low quality based on risk of bias, inconsistency, indirectness, imprecision, and publication bias [27]. The assessment of bias related to small-study effects was performed via visual assessment of funnel plots. While Egger’s regression and Begg’s rank correlation tests were employed to quantify asymmetry, results were interpreted with caution, given the known limitations of these tests when applied to proportional data with low event rates [27].

Results

Study Selection

**Figure 1:**
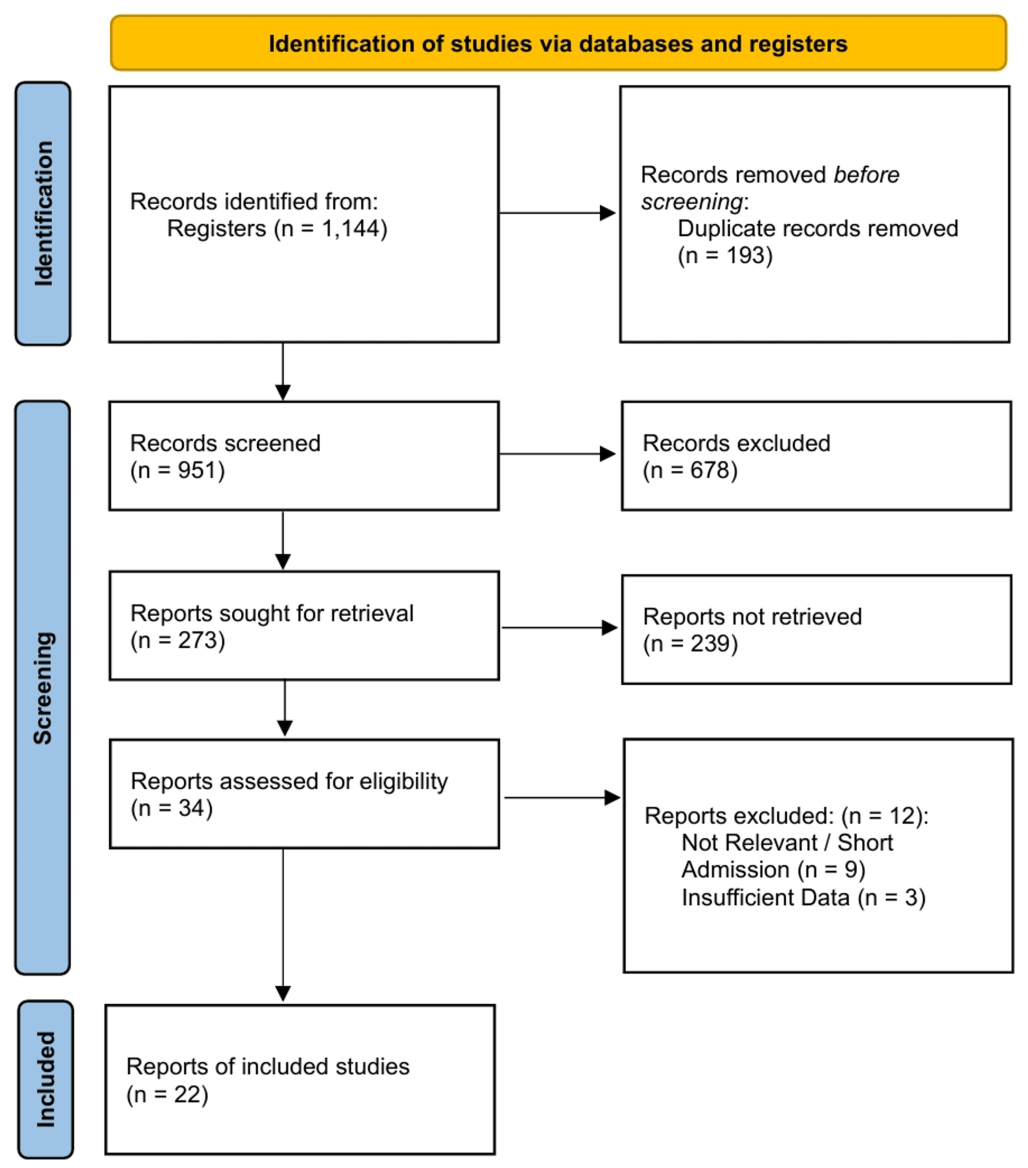
PRISMA 2020 flow diagram for study selection. PRISMA: Preferred Reporting Items for Systematic Reviews and Meta-Analyses

The initial electronic and manual search strategies yielded 1,144 records. After the removal of 193 duplicate records, 951 unique articles were screened based on their titles and abstracts. A total of 678 records were excluded at this stage, and 273 reports were retrieved for full-text evaluation. Of these, 34 reports were assessed for eligibility. Twelve reports were excluded for being irrelevant, representing short admissions, or containing insufficient data for extraction. Twenty-two studies met the inclusion criteria and were included in the qualitative and quantitative analyses [28-49]. The study selection process is detailed in the PRISMA flowchart (Figure 1).

Characteristics of Included Studies and Risk of Bias

The 22 included studies comprised seven RCTs and 15 observational (one prospective and 14 retrospective) cohort studies published between 2000 and 2025. A total of 6,860 endosseous implants in patients who underwent maxillary sinus floor augmentation were analyzed. The mean follow-up duration across the studies ranged from 0.5 to 15 years. The surgical interventions included lateral window, crestal (osteotome), and mixed approaches. The characteristics of the included studies are summarized in Table 1.

**Table 1:** Characteristics of included studies (n=22). RCT: randomized controlled trial

Studies	Year	Design	Implants (n)	Follow-up (years, mean)	Technique	Failures/total	Risk of bias
Ha et al. [28]	2020	Retrospective	202	10.0	Lateral	8/202	Moderate
Zhang et al. [29]	2024	Retrospective	760	5.8	Lateral	15/760	Moderate
Olson et al. [30]	2000	Prospective	120	3.2	Lateral	3/120	Moderate
Hashem et al. [31]	2023	RCT	21	0.5	Crestal	0/21	Low
Jamcoski et al. [32]	2023	Retrospective	757	15.0	Lateral	21/757	Moderate
Krennmair et al. [33]	2019	Prospective	267	5.0	Lateral	2/267	Low
Liu et al. [34]	2019	RCT	75	1.0	Crestal	0/75	Low
Pichotano et al. [35]	2019	RCT	38	1.0	Lateral	0/38	Low
Carmagnola et al. [36]	2024	RCT	57	7.0	Mixed	5/57	Low
Guljé et al. [37]	2014	RCT	41	1.0	Mixed	0/41	Low
Hallman et al. [38]	2005	Prospective	108	3.0	Lateral	15/108	Moderate
Jue et al. [39]	2025	RCT	198	1.0	Mixed	15/198	Low
Jurisic et al. [40]	2008	Retrospective	80	3.0	Mixed	0/80	Moderate
Tetsch et al. [41]	2010	Retrospective	2,190	15.0	Mixed	62/2,190	Moderate
Erdem et al. [42]	2016	Retrospective	58	3.0	Lateral	1/58	Moderate
Hao et al. [43]	2021	Retrospective	93	5.0	Mixed	5/93	Moderate
Khouly et al. [44]	2017	Retrospective	217	7.2	Lateral	22/217	Moderate
Kim et al. [45]	2013	Retrospective	643	3.0	Mixed	69/643	Moderate
Kim et al. [46]	2020	Retrospective	395	10.0	Lateral	13/395	Moderate
Lie et al. [47]	2021	RCT	59	6.0	Mixed	5/59	Low
Sağlanmak et al. [48]	2025	Retrospective	289	12.4	Lateral	19/289	Moderate
Uckan et al. [49]	2011	Retrospective	192	2.8	Mixed	1/192	Moderate

**Figure 2:**
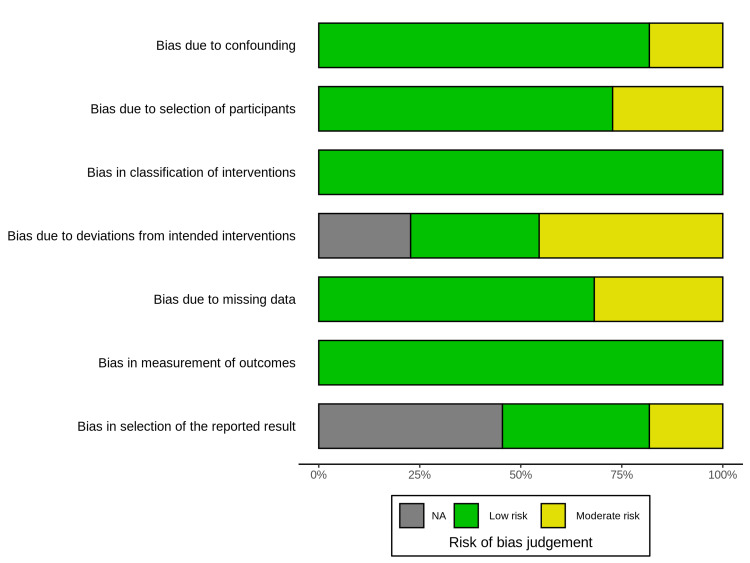
Risk of bias summary.

**Figure 3:**
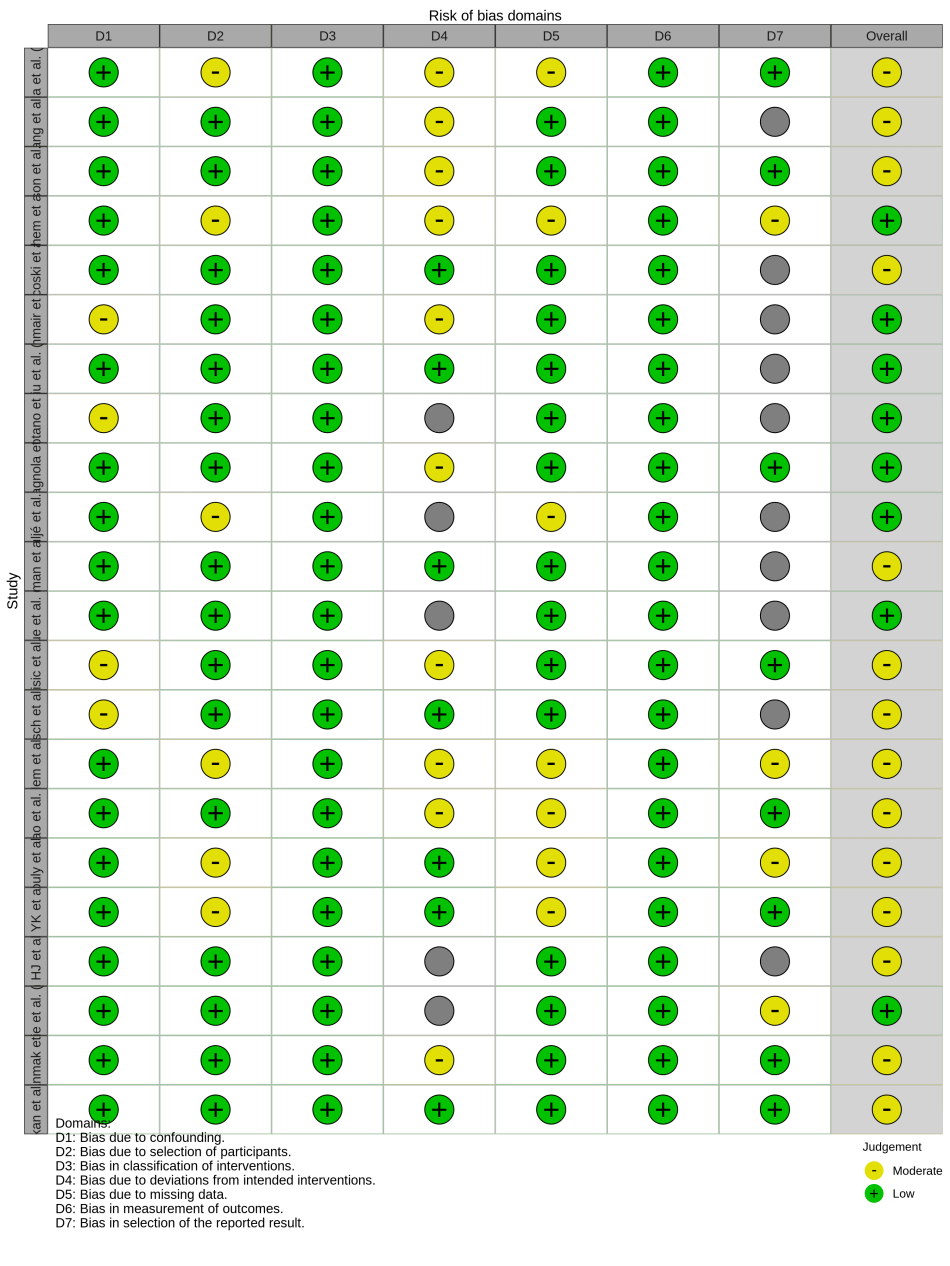
Risk of bias traffic light plot.

The methodological quality was assessed using the RoB 2 tool for RCTs and the Risk Of Bias In Non-randomized Studies of Interventions tool for observational studies. The risk of bias summary indicates that the most prevalent concerns were related to "bias due to missing data" and "bias in selection of the reported result," where a notable proportion of studies were judged to be at moderate risk (Figure 2). The traffic light plot provides a detailed judgment for each individual study across all assessed domains, revealing a generally low-to-moderate risk of bias across the evidence (Figure 3).

Meta-Analysis of Implant Survival (Primary Outcome)

**Figure 4:**
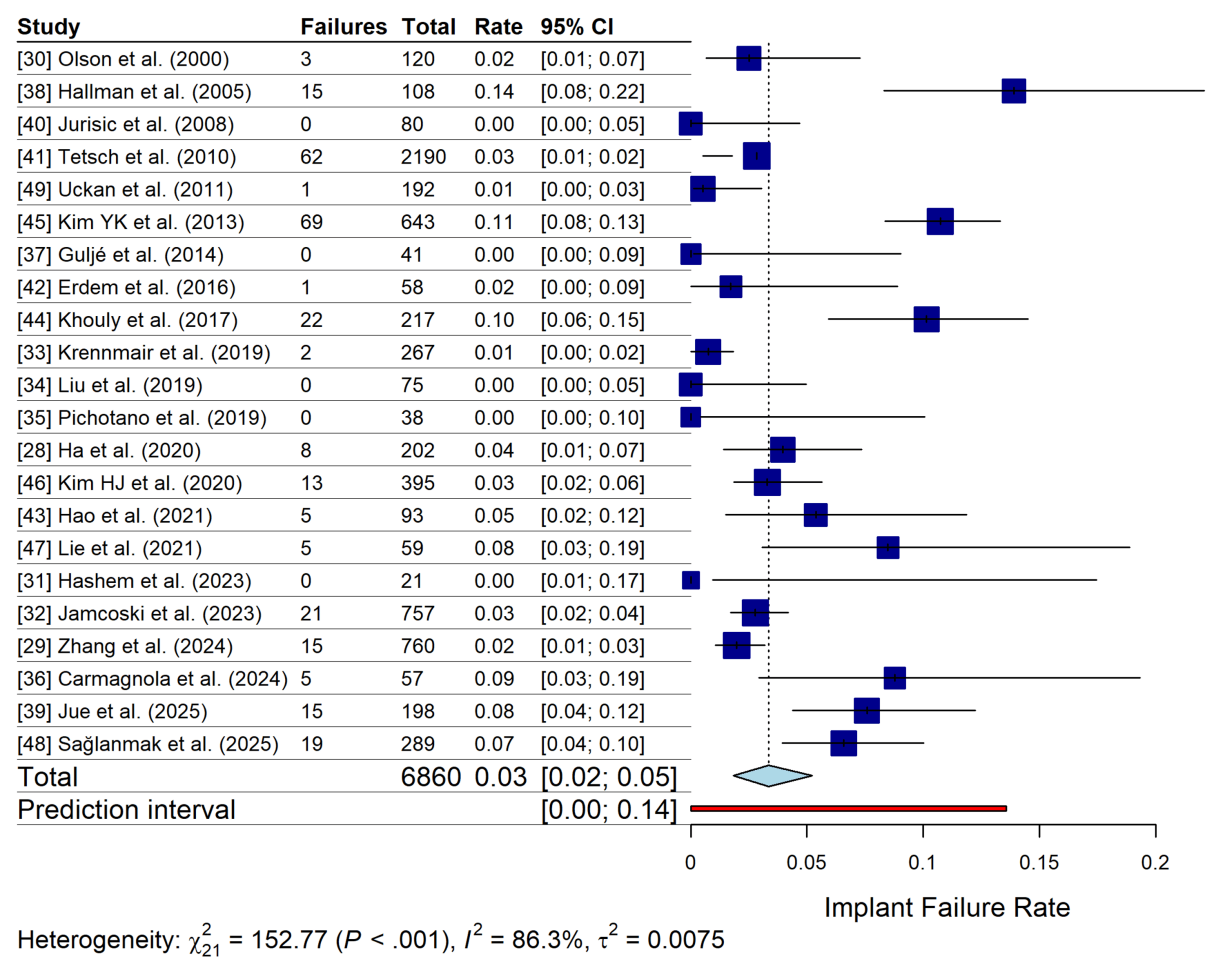
Forest plot of implant failure rates. Random-effects meta-analysis of 22 studies showing the pooled implant failure rate. Squares represent the effect estimate of each study, with size proportional to study weight. The diamond represents the overall pooled estimate. The red bar indicates the 95% prediction interval.

A total of 22 studies reporting on 6,860 implants were included in the meta-analysis for the primary outcome of implant failure rate. The pooled implant failure rate, calculated using a random-effects model with Freeman-Tukey double arcsine transformation and Hartung-Knapp adjustment, was 3% (95% CI: 2-5%). Substantial heterogeneity was observed among the studies (I^2^=86.3%; τ^2^=0.0075; p<0.001). The 95% prediction interval ranged from 0% to 14%, indicating that a future study could report a failure rate within this range. The forest plot of implant failure is presented in Figure 4.

Meta-Analysis of Marginal Bone Loss (Secondary Outcome)

**Figure 5:**
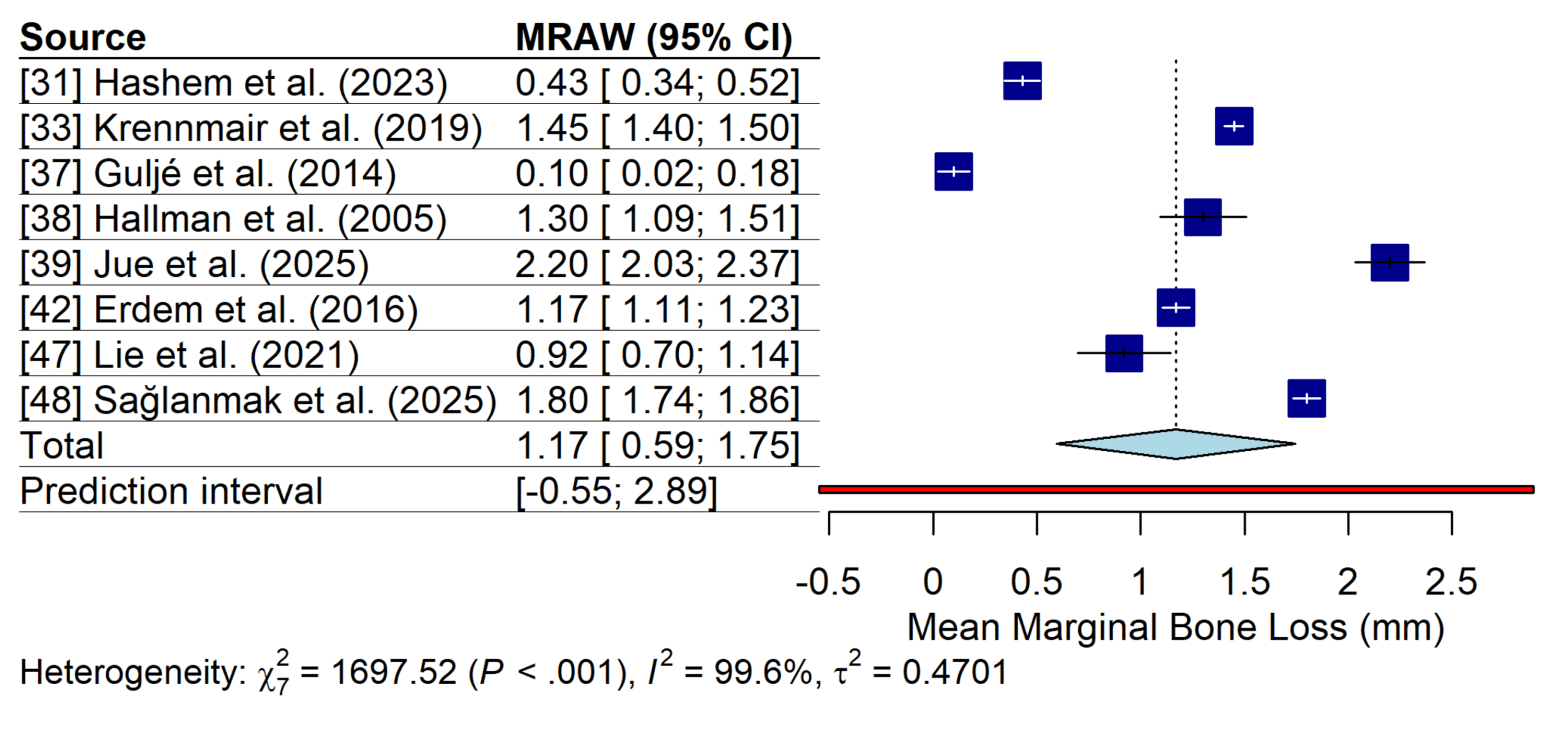
Forest plot of MBL. Random-effects meta-analysis of nine studies showing the pooled mean MBL in millimeters. MBL: marginal bone loss; MRAW: mean radiographic alveolar width

Nine studies provided data suitable for the meta-analysis of marginal bone loss (MBL). The pooled mean marginal bone loss was 1.17 mm (95% CI: 0.59-1.75 mm). The analysis revealed extreme heterogeneity among the studies (I^2^=99.6%; p<0.001). The prediction interval was wide (-0.55 to 2.89 mm), reflecting a high level of inconsistency across studies (Figure 5).

Subgroup Analysis and Meta-Regression

**Figure 6:**
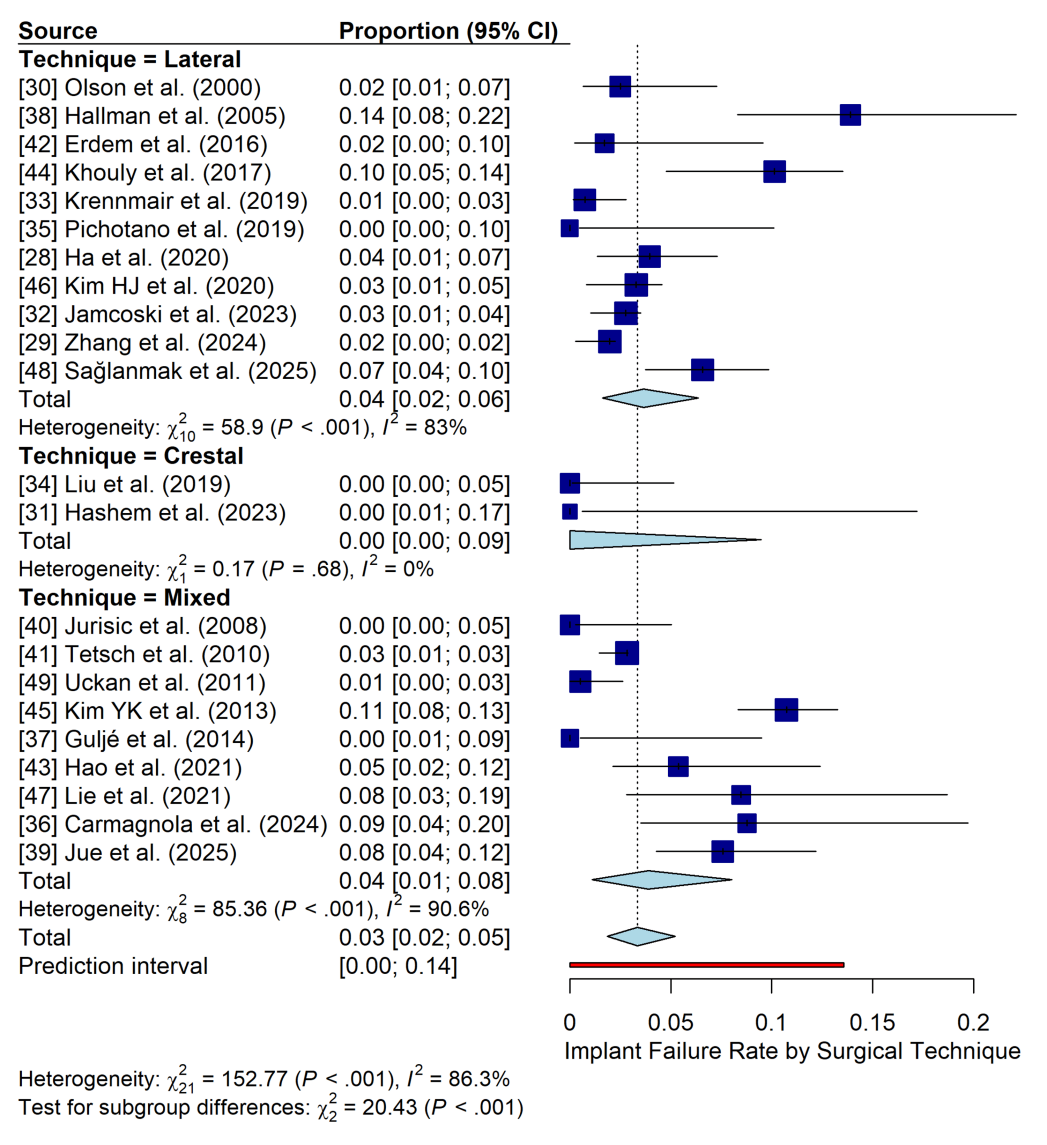
Subgroup analysis of implant failure rates by surgical technique. Forest plot showing the pooled failure rates for lateral, crestal, and mixed approach subgroups. The test for subgroup differences is also presented.

To investigate the high heterogeneity in implant failure rates, a subgroup analysis based on the primary surgical technique was performed (Figure 6). The lateral approach subgroup (12 studies) showed a pooled failure rate of 4% (95% CI: 2-6%; I^2^=83%). The crestal approach subgroup (three studies) had a pooled failure rate of 0% (95% CI: 0-0%; I^2^=0%). The mixed approach subgroup (seven studies) showed a pooled failure rate of 4% (95% CI: 1-8%; I^2^=90.6%). The difference between these subgroups was statistically significant (p<0.001), with the crestal approach demonstrating significantly lower failure rates than the other approaches.

**Figure 7:**
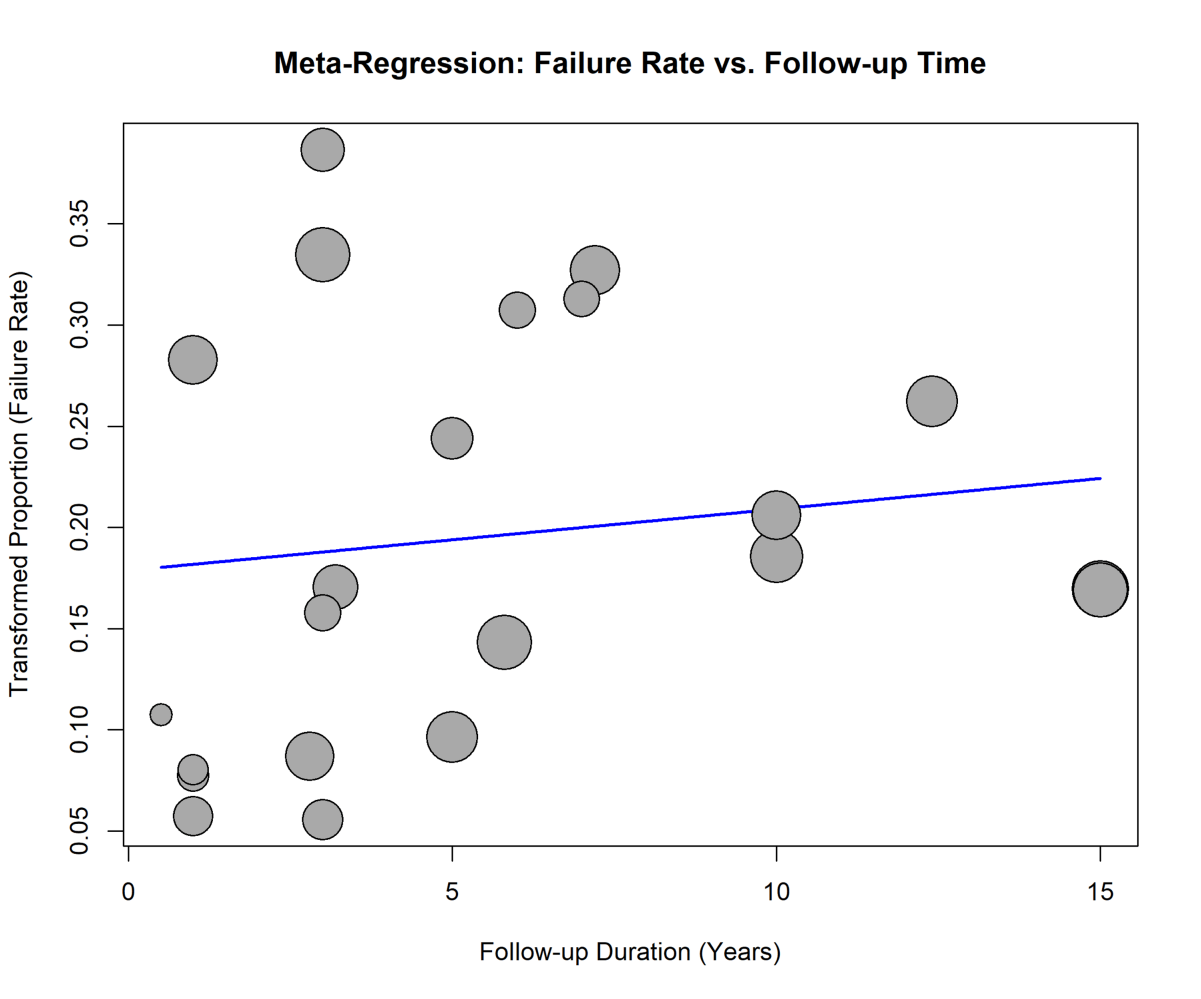
Meta-regression of implant failure rate vs. follow-up duration. Bubble plot illustrating the relationship between the transformed proportion of implant failures and the mean follow-up time in years. The size of each bubble is proportional to the study's precision.

Meta-regression analysis was conducted to assess the influence of follow-up duration on implant failure (Figure 7). The analysis revealed a non-significant positive trend, suggesting that a longer follow-up duration was not statistically associated with an increase in implant failure rates across the included studies (slope=0.003, p=0.76).

Publication Bias

**Figure 8:**
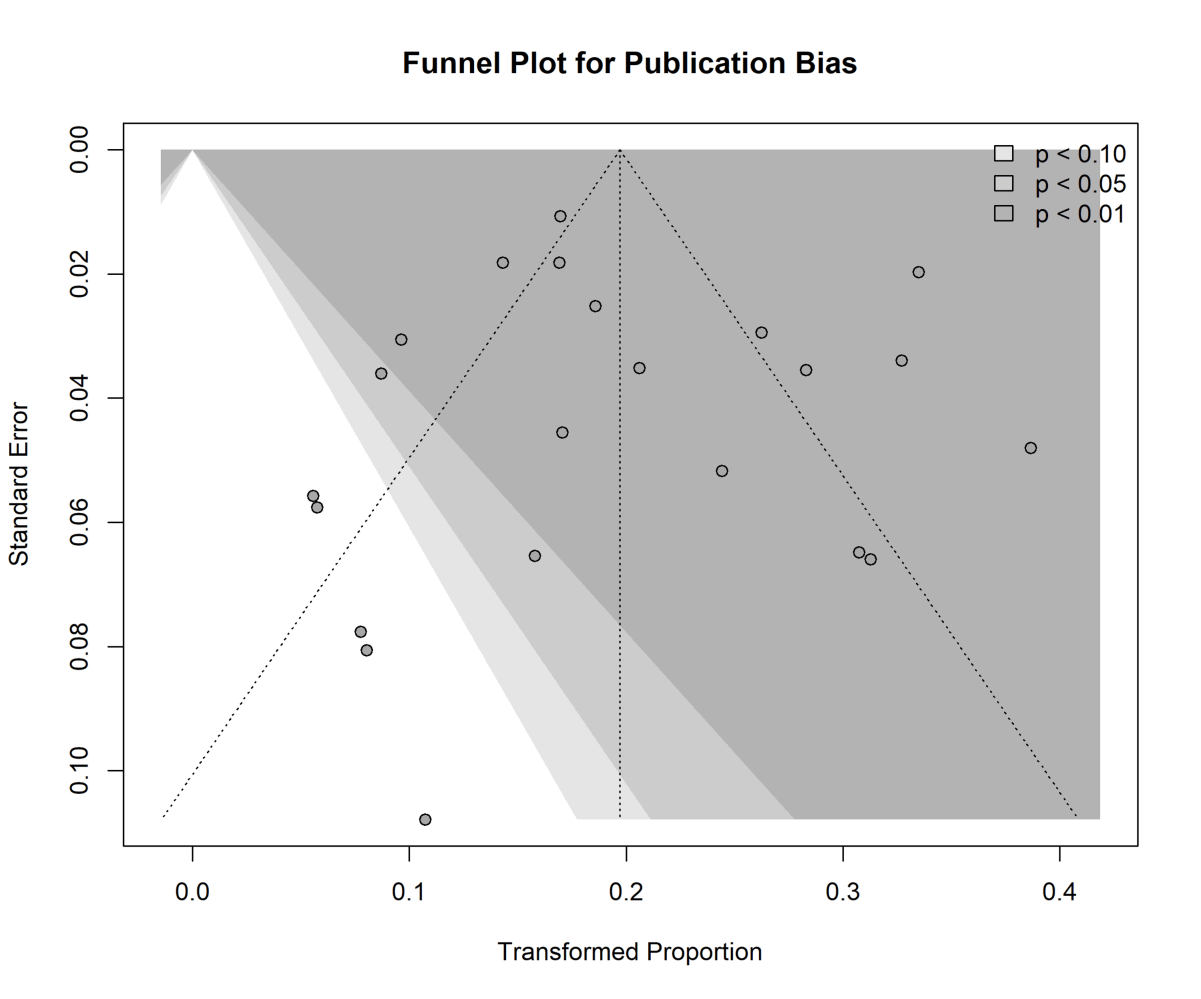
Funnel plot for the assessment of publication bias. A plot of the transformed proportion of implant failures against the standard error. The shaded regions represent the 90%, 95%, and 99% confidence intervals.

Visual inspection of the funnel plot for implant failure revealed a relatively symmetrical distribution of studies around the pooled effect estimate (Figure 8). This observation was supported by formal statistical testing, with neither Egger’s regression test (p=0.76) nor Begg’s rank correlation test (p=0.89) indicating significant evidence of small-study effects or publication bias.

Certainty of the Evidence (GRADE)

The certainty of the evidence for primary (implant failure) and key secondary (marginal bone loss) outcomes were assessed using the GRADE approach [27]. The overall body of evidence started at a "high" certainty level due to the inclusion of multiple RCTs. We considered the following five domains for downgrading the quality of evidence: risk of bias, inconsistency, indirectness, imprecision, and publication bias. For the implant failure outcome, the evidence was downgraded by two levels to low certainty.

Risk of bias (-1): Downgraded one level for "serious" risk of bias. Although seven studies were RCTs, most of the evidence base (15 studies) consisted of observational studies, most of which were judged to be at moderate risk of bias due to potential confounding and selection bias.

Inconsistency (-1): Downgraded one level for "serious" inconsistency. The meta-analysis revealed substantial statistical heterogeneity (I^2^=86.3%) that was not fully explained by subgroup or meta-regression analyses. The wide prediction interval (0-14%) further indicates that the true effect in a new study could be substantially different from the pooled estimate.

For the marginal bone loss outcome, the evidence was also downgraded by two levels to low certainty.

Risk of bias (-1): Downgraded for the same reasons as for the primary outcome.

Inconsistency (-1): Downgraded for "very serious" inconsistency, as evidenced by the extremely high statistical heterogeneity (I^2^=99.6%) and a wide prediction interval that crossed the line of no effect.

There were no serious concerns regarding indirectness, imprecision (due to the large sample size and narrow CIs for the primary outcome), or publication bias (supported by funnel plots and statistical tests). The summary of findings is presented in Table 2.

**Table 2:** GRADE summary of findings for key outcomes. GRADE working group grades of evidence are as follows: high certainty, moderate certainty, low certainty, and very low certainty. The two filled circles (⊕⊕) and two empty circles (◯◯) visually signify that the evidence is of low certainty because it has two major limitations. GRADE: Grading of Recommendations, Assessment, Development, and Evaluations

Outcomes	Anticipated absolute effects (95% CI) per 1,000 implants	Participants, n (studies)	Certainty of the evidence (GRADE)	Comments
Implant failure	30 per 1,000 (20-50 per 1,000)	6,860 implants (22 studies)	⊕⊕◯◯ Low	Downgraded two levels: one for serious risk of bias (predominance of observational studies) and one for serious inconsistency (I^2^=86.3%; wide prediction interval).
Marginal bone loss	Mean 1.17 mm (95% CI: 0.59-1.75 mm)	4,502 implants (9 studies)	⊕⊕◯◯ Low	Downgraded two levels: one for serious risk of bias and one for very serious inconsistency (I^2^=99.6%). The true effect is likely to be substantially different.

Discussion

This systematic review and meta-analysis, encompassing 22 studies and 6,860 implants, confirms that maxillary sinus floor augmentation is a highly predictable and successful procedure for the rehabilitation of the atrophic posterior maxilla. The primary finding of this analysis is a pooled implant failure rate of 3% (95% CI: 2-5%), corresponding to a cumulative survival rate of 97%. Subgroup analysis revealed that the surgical technique is a significant moderator of outcomes, with implants placed via a crestal approach demonstrating a significantly lower failure rate than those placed with a lateral window approach (p<0.001). The pooled mean marginal bone loss was 1.17 mm (95% CI: 0.59-1.75 mm), a value considered clinically acceptable. However, despite these positive outcomes, the certainty of the evidence for both implant survival and marginal bone loss was graded as low, due to the significant risk of bias from the predominance of observational studies and substantial inconsistency across study results.

The 97% implant survival rate observed in this analysis aligns with the studies by Duttenhoefer et al. and Moraschini et al. [4,8]. However, unlike previous reviews, which relied on older data, our analysis integrates 22 studies up to 2025, confirming that high survival rates persist even as surgical indications expand to include more severe atrophy and graftless protocols. While Hallman et al. reported a higher failure rate (14%), this remains an outlier attributable to the use of machined-surface implants, which are no longer the clinical standard [38].

The mean marginal bone loss of 1.17 mm is consistent with the established biological principles of bone remodeling following prosthetic loading. However, the extreme heterogeneity observed (I^2^=99.6%) underscores a critical challenge in the literature as follows: the lack of a standardized methodology for MBL measurement. This variability stems from differences in radiographic techniques (panoramic vs. periapical), follow-up intervals, and the diverse implant-abutment connections used in the included studies. The findings of Krennmair et al., who reported a mean MBL of 1.45 mm at five years, are consistent with this expected range, but the wide prediction interval in our analysis (-0.55 to 2.89 mm) indicates that the MBL in any future study could vary substantially [33].

The most significant finding from the moderator analyses was the superior performance of the crestal (osteotome) approach compared with the lateral window technique. The pooled failure rate for the crestal subgroup was nearly zero, which was a statistically significant difference. However, this result should be interpreted with caution. The crestal approach is indicated for cases with more favorable residual bone height (RBH), typically ≥5 mm, as seen in the studies by Hashem et al. and Liu et al. [31,34], whereas the lateral window technique is reserved for more challenging scenarios with severe atrophy (RBH <4 mm), as documented by Kim et al. [46]. Therefore, the observed difference in survival is confounded by case selection bias, where the crestal technique is applied to anatomically less demanding situations with better intrinsic prognosis.

The meta-regression analysis did not find a statistically significant association between follow-up duration and implant failure, which suggests that most implant failures in augmented sinuses are early failures, occurring within the first few years of function, with the survival rate stabilizing thereafter. This is consistent with the findings of Zhang et al. and aligns with the concept that failures are primarily related to compromised osseointegration rather than late-stage peri-implantitis [29].

Strengths

The primary strength of this review is its comprehensive scope, synthesizing data from more than 6,800 implants across a 25-year publication period. Methodologically, adherence to PRISMA guidelines, prospective registration, dual-reviewer assessment, and the application of robust statistical methods, including the Freeman-Tukey transformation for zero-event studies and the Hartung-Knapp adjustment for variance, enhanced the validity of the findings. The use of both RoB 2 and ROBINS-I tools provided a design-specific and rigorous appraisal of the bias.

Limitations

The principal limitation of this study is the substantial heterogeneity across the included studies. This inconsistency arises from the mix of study designs (RCTs vs. observational studies), significant variations in follow-up duration (0.5-15 years), and a lack of standardized protocols for surgical technique, graft material selection, and outcome measurement. This heterogeneity was the primary reason for downgrading the certainty of evidence to "low" using the GRADE framework. Furthermore, most retrospective studies, which constitute the bulk of the evidence, exhibit a moderate risk of bias, thereby limiting causal inference.

Clinical implications

This review confirms that maxillary sinus floor augmentation is a safe and highly predictable procedure for enabling implant therapy, with an overall implant survival rate of >97%. The choice between the lateral and crestal approaches should be guided by the residual bone height rather than the perceived superiority of one technique over the other. In cases with adequate RBH (≥5 mm), a crestal approach offers a less invasive and highly successful option. In severely atrophic cases, the lateral window remains the standard of care, with excellent long-term outcomes.

Future directions

Future research should prioritize methodological standardization. High-quality, long-term RCTs are needed to compare lateral and crestal techniques in homogenous patient populations (e.g., all patients with 4-6 mm RBH). The establishment and adoption of a core outcome set (COS) for sinus augmentation studies, mandating standardized reporting of RBH and MBL measurement techniques and complication definitions, are crucial to reduce heterogeneity and enable more powerful future meta-analyses.

## Conclusions

This systematic review and meta-analysis demonstrated that maxillary sinus floor augmentation is a highly reliable procedure that facilitates implant survival rates exceeding 97% in the long term. Although the crestal approach is associated with fewer implant failures, this finding is likely confounded by patient selection. The overall certainty of the evidence was low, underscoring the need for future research with greater methodological rigor and standardized reporting to refine clinical guidelines.
